# Effects of Intraoperative Prone Versus Supine Positioning on Postoperative Delirium

**DOI:** 10.3390/geriatrics11020048

**Published:** 2026-04-19

**Authors:** Theresa E. Hering, Maria Wittmann, Vera Guttenthaler, Robert Pflugmacher, Rudolf Hering

**Affiliations:** 1 Department of Orthopedic and Trauma Surgery, Mechernich District Hospital, 53894 Mechernich, Germany; 2 Department of Orthopedic and Trauma Surgery, Garmisch-Partenkirchen Hospital, 82467 Garmisch-Partenkirchen, Germany; 3 Department of Anesthesiology and Intensive Care Medicine, University of Bonn, 53127 Bonn, Germany; maria.wittmann@ukbonn.de (M.W.); vera.guttenthaler@ukbonn.de (V.G.); 4 Department of Spine Surgery, Mechernich District Hospital, 53894 Mechernich, Germany; 5 Department of Anesthesiology and Intensive Care Medicine, Mechernich District Hospital, 53894 Mechernich, Germany

**Keywords:** postoperative delirium, geriatric surgery, prone position, risk factors

## Abstract

**Background**: Postoperative delirium (POD) is a common complication in geriatric patients. This prospective cohort study evaluated a possible influence of intraoperative positioning on the occurrence of POD, as intraoperative prone positioning could affect cerebral perfusion. **Methods:** We included 760 patients of ≥60 years scheduled for elective surgery in prone or supine positions. The primary outcome was POD incidence on the first five days after surgery, assessed via 3D-Confusion Assessment Method (3-D CAM) or Confusion Assessment Method for Intensive Care Units (CAM-ICU). Preoperative assessments included the American Society of Anesthesiologists (ASA) and New York Heart Association (NYHA) classifications as well as short screenings for the cognitive (modified Montreal Cognitive Assessment (MoCA)) and self-care status of the patient. Secondary outcomes were length of hospital stay (LOS) and mortality rates. **Results**: Postoperative delirium rates were similar in prone and supine patients (7.6% vs. 5.5%; *p* = 0.31), and logistic regression analysis revealed no association of intraoperative prone positioning with POD (odds ratio 1.42 (95% CI 0.68–2.92; *p* = 0.342)). The overall incidence of POD was 6.1% and was associated with older age (81.5 (CI 76.2–84.8) vs. 72.0 (CI 67.0–79.0) years; *p* < 0.01), higher ASA and NHYA classifications, lower preoperative modified MoCA, reduced independence in self-care (*p* < 0.001, respectively), and longer incision-to-suture times (107.0 (CI 73.0–173.0) vs. 85.0 (CI 60.0–130.0) minutes; *p* < 0.01). Postoperative delirium resulted in longer LOS (14.5 (CI 9.0–27.0) vs. 7.0 (CI 4.0–9.0) days; *p* < 0.001), and increased mortality (13.0% vs. 1.7%; *p* < 0.001). **Conclusions:** Intraoperative prone positioning was not associated with POD in patients aged 60 years or older (OR 1.42; CI 0.68–2.92; *p* < 0.340), and LOS and mortality as secondary outcome parameters were also similar in patients after prone and supine surgery. Future studies assessing additional and possible confounding factors and intraoperative systemic and regional hemodynamics and oxygenation are needed to verify this result and to evaluate cerebral hypoperfusion as a possible mechanism of POD.

## 1. Introduction

Postoperative delirium (POD) is a common postoperative complication in older patients and is defined as an acute, fluctuating disturbance of consciousness, attention, and perception that affects memory, language, and temporal and spatial orientation [1,2]. Postoperative delirium can occur as a hyperactive form with agitation and restlessness, as a hypoactive form with apathy and lethargy, or as a mixed form [3]. The incidence of POD varies considerably in the literature, partly due to the inconsistent use of diagnostic instruments [4].

Postoperative delirium can significantly worsen the prognosis of affected patients, with the main issues being temporary or irreversible cognitive dysfunction and functional limitations, the need for care, and loss of independence, up to and including increased mortality [5,6,7]. The etiology of POD has not yet been fully elucidated, but it is assumed to be multifactorial [8,9]. Depth of anesthesia, intraoperative blood loss, and duration of surgery have been identified as perioperative risk factors, but the role of intraoperative positioning as a possible independent POD risk factor has not been investigated explicitly [10,11,12]. However, intraoperative prone positioning can lead to relevant increases in intrathoracic and intra-abdominal pressure with significant restrictions on ventricular compliance, cardiac preload, cardiac output, and arterial blood pressure, resulting in a drop in cerebral blood flow and reduced oxygen supply, which, as a consequence, may increase the risk of POD [13,14,15,16,17]. Previous studies examining cerebral oxygenation changes during prone positioning [17,18,19,20] demonstrated reduced [17,18] or temporarily reduced cerebral oxygenation [19,20]. In a study linking cerebral oxygen desaturation to POD, Wang et al. found a lower incidence of POD in patients who received lung-protective ventilation in the prone position during spinal surgery compared to conventional mechanical ventilation [21]. In this study, lung-protective ventilation was associated with reduced systemic inflammation, reduced tissue oxygen consumption, as well as improved cerebral oxygen metabolism. In addition, Kim et al. found the total duration of regional SO_2_ to be an independent risk factor for postoperative cognitive dysfunction in patients aged 65 years or older who underwent lumbar spine surgery in the prone position [22].

However, to the best of our knowledge, this is the first study that directly investigated the association of intraoperative prone versus supine positioning on the incidence of POD.

## 2. Materials and Methods

### 2.1. Study Population

The patients included in this study were a subset of a multicentric external validation study of the PROPDESC score (Pre-Operative Prediction of Postoperative Delirium by Appropriate Screening score) [23]. In accordance with the specifications of that study, patients aged 60 years and older scheduled for elective surgery of at least one hour and a hospital stay of at least one night who provided written informed consent for study participation were included. Exclusion criteria were preoperative cognitive deficits, dementia, insufficient knowledge of the German language, and emergency or intracranial surgery [23].

### 2.2. Study Design

Ethics vote was provided by the Ethics committee of the Medical Faculty of the Rheinische Friedrich-Wilhelms-University of Bonn (136/22). The investigation was conducted at Mechernich District Hospital, an academic teaching hospital of the University of Bonn, Germany, as a prospective cohort study [23].

During preanesthesia evaluation, the patients baseline characteristics were recorded by trained physicians, which included age, gender, body mass index (BMI), American Society of Anesthesiologists (ASA) Physical Status Classification, and New York Heart Association Classification (NYHA). To classify the surgical risk of the planned surgery, the original Johns Hopkins surgical classification was used in a modified form as a three-level surgical classification in accordance with the guidelines of the European Society of Anesthesiology (ESA) (I–II = low-risk, III = intermediate-risk, IV–V = high-risk surgery) [24,25].

In addition, a short preoperative cognitive screening was performed, which consisted of two tasks from the Montreal Cognitive Assessment (MoCA), a subtraction task and a sentence repetition task [26]. Patients were also asked to answer a question about self-care taken from the EQ-5D-5L [27].

Anesthesia was administered according to clinical requirements and without further instructions to the respective anesthesiologists. Balanced anesthesia (sevoflurane or isoflurane as inhalational agents and fentanyl or sufentanil as analgesics) or total intravenous anesthesia (Propofol and remifentanil) using intubation or laryngeal mask techniques were used, as well as a standard hemodynamic and transfusion management. Patients receiving spinal or peripheral regional anesthesia in combination with general anesthesia were classified as patients who received general anesthesia.

Intraoperative positioning was performed in accordance with established positioning standards. During prone surgery, positioning cushions were used to avoid excessive pressure on the abdomen and allow unrestricted ventilation, venous return to the heart, and cardiac output. The head was positioned in a neutral position in a headrest (Prone Face Plus, Nordiska, Gummersbach, Germany), allowing pressure-free positioning of the eyes, nose, chin, larynx, and soft tissues.

Postoperatively, patients were monitored in the recovery room in accordance with the clinic standard operating procedures until sufficient alertness, stable cardiopulmonary function (SpO_2_ > 90% in room air or reaching the preoperative level), and a low level of pain were established.

### 2.3. Outcome Variables

Delirium tests were conducted by trained physicians, started on the first postoperative day, and was performed once daily on five consecutive days or until the day of discharge, whichever occurred first. Testing in the intensive care unit (ICU) started when the patient reached a Richmond Agitation and Sedation Scale score of minus three or higher, which assesses the patient as moderately sedated but responsive.

For detection of POD as the primary outcome parameter, we used the 3D Confusion Assessment Method (3 min diagnostic interview for CAM-defined delirium; 3D-CAM) in the normal ward or intermediate care station [28] and the Confusion Assessment Method for ICU (CAM-ICU) in the ICU [29,30].

Patient interviews were conducted between 8:00 a.m. and 4:00 p.m. In addition, the attending nurses were interviewed and the entries in the patient chart over the previous 24 h were reviewed to detect potential POD at evening or night times.

Length of hospital stay (LOS) and survival status as secondary outcome parameters were recorded on the day of discharge.

### 2.4. Statistical Analyses

A power analysis was performed to estimate the number of cases required to answer the primary question. Assuming an α error of 0.05, a power of 80%, and an effect size of 0.02, a sample size of 752 patients was calculated in order to determine the influence of possible predictors on the primary endpoint parameter POD using regression analysis [31]. For univariate data analysis, metric variables were specified as median and interquartile range and compared using the two-tailed Mann–Whitney U test, while nominal and ordinal variables were specified as frequency per total number and as percentage (%) and compared using the two-tailed Fisher’s exact test or Chi-squared test, respectively.

In addition to prone position, the covariates age, gender, ASA and NYHA class, surgical risk, incision-to-suture time, and a modified MoCA score were selected as possible confounding factors which influence was to be controlled for in the logistic regression analysis. Spearman correlation coefficients were calculated to detect collinearities between the covariates.

A correlation coefficient, R > 0.3 between individual covariates, was considered relevant for further statistical analysis. For submodel testing of the statistical significance of the final regression model compared to the simple (prone position only) and full models (all factors deemed relevant: prone position, age, gender, ASA class, NYHA class, surgical risk, modified MoCA score, and incision-to-suture time), deviance tests for generalized linear models were performed. Odds ratios were reported with a 95% confidence interval (CI).

## 3. Results

### 3.1. Patients

**Figure 1:**
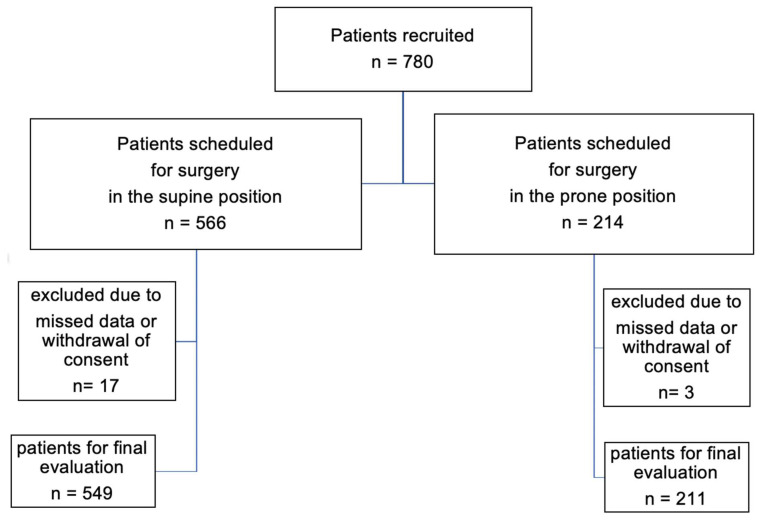
Patient assignment to the intraoperative supine and prone position groups.

Of the 780 patients included in the external PROPDESC validation study at Mechernich District Hospital between February 2023 and March 2024, 566 (72.6%) patients underwent surgery in the supine position and 214 (27.4%) in prone position. Due to withdrawal of consent or incomplete data, 17 (3.0%) patients of the supine position group and 3 (1.4%) patients of the prone position group had to be excluded. Thus, data of 760 patients, 211 (28%) in the prone and 549 (72%) in the supine group were analyzed (Figure 1).

### 3.2. Comparison of Patients After Surgery in Prone and Supine Position

Preoperative data of patients after surgery in the prone or supine position were comparable with respect to age, BMI, and ASA and NYHA classification, the results of the preoperative cognitive screening, and their self-care status (Table 1). Patients who underwent surgery in the prone position were almost exclusively operated on the spine; thus, a statistical evaluation with regard to the surgical area was deliberately omitted (Table 1). Spinal surgery is predominantly a medium-risk surgery. This led to a significant difference in the surgical risk between patients who underwent surgery in supine and prone positions (Table 1). In addition, the duration of surgery in the prone position was longer than that in supine position (Table 1).

The incidence and duration of POD, LOS, as well as mortality rates did not differ significantly between the supine and prone groups (Table 2).

Table 3 shows the results of the logistic regression analysis. Considering the relevant influencing factors determined for the final regression model age, ASA class, modified MoCA score, and incision-to-suture time, there was no statistical evidence for an influence of intraoperative prone positioning on the development of POD (odds ratio of 1.42 (95% CI 0.68–2.92, *p* = 0.342).

### 3.3. Comparison of Patients with and Without Postoperative Delirium

The overall incidence of POD was 46 of 760 patients (6.1%) (Table 4). Patients with POD were older, had higher ASA and NYHA classifications, lower BMI, scored lower in the preoperative modified MoCA screening, and were more dependent regarding their daily self-care. Additionally, they had to undergo longer surgeries.

Patients with POD had longer LOS and higher mortality rates after both intraoperative supine and prone positioning (Table 5).

## 4. Discussion

This study demonstrated that intraoperative prone positioning is not associated with a higher incidence of POD compared to supine positioning, regardless of other influencing factors.

The incidence of POD in our study was 6.1%. This relatively low incidence could be explained by the fact that our study sample did not include cardio-surgical patients and we excluded patients with intracranial surgery as well as emergency cases [32,33]. In line with former studies, advanced age, pre-existing cognitive impairment, and reduced ability to cope with everyday activities were associated with a significantly higher risk of developing POD even in our patients [34,35,36,37]. Furthermore, the results of this study confirm previous investigations that the probability of developing POD increases with reduced cardiac capacity, higher anesthetic risk, a longer incision-to-suture time, and a lower BMI [38,39,40,41,42]. Importantly, in accordance with earlier studies, mortality was also significantly higher in our patients with POD [5]. Our results confirm that POD can be associated with a very serious, potentially persistent, and even life-threatening deterioration in health.

In our study, the incidence of POD was similar after surgery in the prone and supine positions, and there was no difference in LOS or mortality between those two patient groups. Most of our patients in the prone group underwent spinal or orthopedic joint surgery. Therefore, these results are clinically relevant, as many operations in the prone position, especially in spinal surgery, involve older, frail, and cognitively impaired or (pre-) dementia patients with an inherently increased risk of POD. In a recent study by Susano et al., 54% of patients older than 70 years undergoing elective spinal surgery were classified as moderately frail, and 24% as frail, and Lee et al. detected previously undiagnosed cognitive impairment in 38% of their patients who were scheduled for elective spinal surgery [43,44].

Patients in prone and supine positions during surgery did not differ in biometric data, ASA and NYHA classification, or preoperative cognitive and physical capabilities, indicating that both groups were comparable. However, the incision-to-suture times for operations in prone position were longer than in supine position, which may be explained by the fact that operations in the prone position were mainly performed for spinal surgery. Although total anesthesia and surgery times were not documented in our study, it can be assumed that they were significantly longer for surgeries performed in the prone position compared to those performed in supine position. This is also confirmed by data from Deiner et al., who observed longer surgery times for spinal surgeries in the prone position compared to supine position surgeries [17]. Although longer surgery times correlate significantly with the incidence of POD in both our and former studies [42], POD rate was not higher after prone position surgeries in our study.

As POD assessment was conducted between 8:00 a.m. and 4:00 p.m, some POD events might have remained unrecognized, even though the attending nursing staff was interviewed and the patient chart was reviewed to minimize this risk. However, hypoactive phases of POD could still have been overlooked in individual cases.

Since cerebral hypoperfusion is likely to promote POD, it is important to preserve stable systemic and regional cerebral hemodynamics during prone positioning [45]. Therefore, we used special positioning cushions in the thorax and pelvic areas to relieve the abdomen to assure unrestricted ventilation, venous return to the heart, and cardiac output. In particular, the head and neck were positioned in a neutral position in a special headrest to avoid any pressure to the soft tissues and vessels of the neck and to enable unaffected cerebral perfusion.

The study has several limitations. First, randomization of patients to the prone or supine group was not possible due to the positioning requirements of the different surgeries. Patients undergoing surgery in the prone position were almost exclusively operated on the spine, whereas there was a significantly greater heterogeneity regarding the surgical procedures in the supine position group. As a result, almost all surgeries in the prone position group were assigned to the medium-surgical-risk class, while all surgical risk classes were represented in the supine position group. Second, although postoperative pain is a well-established risk factor for POD, and pain intensity and analgesic requirements may differ between spinal surgery in prone position and the heterogeneous procedurces performed in supine position, postoperative pain assessment and postoperative analgesic management were not adjusted for in the analysis. Third, specific cardiovascular comorbidities such as hypertensive disease, prior stroke, diabetes mellitus, or atrial fibrillation, which can significantly influence cerebral perfusion, were not explicitly documented as baseline characteristics, and neither systemic nor regional hemodynamic and oxygenation data were assessed. Thus, the potential impact of cerebral hypoperfusion linking prone positioning to POD risk remains speculative.

Fourth, when interpreting our results, it should also be noted that we intended to study the effect of intraoperative prone versus supine positioning on the POD rate in a common clinical setting. Therefore, the effect of intraoperative prone versus supine positioning on the POD rate was evaluated while leaving anesthesiologic, hemodynamic, and transfusion management to the discretion of the attending anesthesiologist, which might have led to additional confounding effects on the results.

## 5. Conclusions

Based on our data, we found no statistically significant association between intraoperative prone positioning and POD in patients age 60 years and older. In addition, LOS and mortality as secondary outcome parameters were also similar in patients after prone and supine surgery.

Our results confirm previous investigations that the probability of developing POD increases with reduced cardiac capacity, higher anesthetic risk, a longer incision-to-suture time, and a lower BMI.

Future studies assessing additional, possibly confounding factors and intraoperative systemic and regional hemodynamics and oxygenation are needed to verify this result and to evaluate cerebral hypoperfusion as a possible mechanism of POD.

## Figures and Tables

**Table 1:** Characteristics of patients undergoing surgery in supine or prone position.

	All Patients (*n* = 760)	Patients Undergoing Surgery in Supine Position (*n* = 549)	Patients Undergoing Surgery in Prone Position (*n* = 211)	*p*-Value
Patient age (years; median (IQR))	73.0 (67.0–80.0)	73.0 (67.0–81.0)	72.0 (67.5–77.5)	0.54
Gender (male/female)	397/363 (52.2%/47.8%)	291/258 (53.0%/47.0%)	106/105 (50.2%/49.8%)	0.52
ASA	I: 40 (5.3%) II: 312 (41.1%) III: 395 (52.0%) IV: 13 (1.7%)	I: 29 (5.3%) II: 210 (38.3%) III: 301 (54.8%) IV: 9 (1.6%)	I: 11 (5.2%) II: 102 (48.3%) III: 94 (44.6%) IV: 4 (1.9%)	0.07
NYHA	I: 464 (61.1%) II: 226 (29.7%) III: 64 (8.4%) IV: 6 (0.8%)	I: 324 (59.0%) II: 174 (31.7%) III: 46 (8.4%) IV: 5 (0.9%)	I: 140 (66.4%) II: 52 (24.6%) III: 18 (8.5%) IV: 1 (0.5%)	0.23
Body mass index (kg/m^2^; median (IQR))	27.0 (24.0–30.5)	27.0 (23.7–30.4)	28.0 (25.0–31.3)	0.16
Modified MoCA: - 0 points - 1 point - 2 points - 3 points - 4 points - 5 points	11 (1.4%) 23 (3.0%) 53 (7.0%) 117 (15.4%) 216 (28.4%) 340 (44.7%)	8 (1.5%) 17 (3.1%) 41 (7.5%) 86 (15.7%) 152 (27.7%) 245 (44.6%)	3 (1.4%) 6 (2.8%) 12 (5.7%) 31 (14.7%) 64 (30.3%) 95 (45.0%)	0.94
Self-care: - No problems - Few problems - Moderate problems - Many problems - Not possible	490 (64.5%) 125 (16.4%) 107 (14.1%) 35 (4.6%) 3 (0.4%)	357 (65.0%) 88 (16.0%) 76 (13.8%) 27 (4.9%) 1 (0.2%)	133 (63.0%) 37 (17.5%) 31 (14.7%) 8 (3.8%) 2 (0.9%)	0.55
Surgical specialty: - Spine - Orthopedic/joint - Head/neck - Vascular - Hepato-biliary - Upper GIT - Lower GIT - Gynecology - Urology/kidney - Other	218 (28.7%) 225 (29.6%) 6 (0.8%) 123 (16.2%) 13 (1.7%) 6 (0.8%) 30 (3.9%) 17 (2.2%) 87 (11.4%) 35 (4.6%)	14 (2.6%) 219 (39.9%) 6 (1.1%) 123 (22.4%) 13 (2.4%) 6 (1.1%) 30 (5.5%) 17 (3.1%) 86 (15.7%) 35 (6.4%)	204 (96.7%) 6 (2.8%) 0 (0.0%) 0 (0.0%) 0 (0.0%) 0 (0.0%) 0 (0.0%) 0 (0.0%) 1 (0.5%) 0 (0.0%)	n.a.
Surgical risk - Low - Intermediate - High	203 (26.7%) 511 (67.2%) 46 (6.1%)	191 (34.8%) 312 (56.8%) 46 (8.4%)	12 (5.7%) 199 (94.3%) 0 (0%)	<0.001
Incision-to-suture-time (minutes; median (IQR))	90.0 (60.0–133.0)	80.0 (54.0–119.0)	120.0 (73.0–180.0)	<0.001

ASA, American Society of Anesthesiologists; GIT, gastrointestinal tract; IQR, interquartile Range; MoCA, Montreal Cognitive Assessment; NYHA, New York Heart Association; POD (postoperative delirium).

**Table 2:** Incidence and duration of postoperative delirium, length of hospital stay, and mortality in patients after supine and prone surgery.

	All Patients (*n* = 760)	Patients Undergoing Surgery in Supine Position (*n* = 549)	Patients Undergoing Surgery in Prone Position (*n* = 211)	*p*-Value
Incidence of POD	46 (6.1%)	30 (5.5%)	16 (7.6%)	0.31
Duration of POD (days; median (IQR))	1.0 (1.0–2.0)	1.0 (1.0–2.0)	1.0 (1.0–2.3)	0.95
LOS (days; median (IQR))	7 (4–10)	7 (4–10)	6 (5–10)	0.81
Mortality rate	18/760 (2.4%)	13/549 (2.4%)	5/211 (2.4%)	1.0

IQR, interquartile range; LOS, length of hospital stay; POD, postoperative delirium.

**Table 3:** Logistic regression analysis of influencing factors.

	Postoperative Delirium		
	Odds Ratio	CI-95%	*p*-Value
Prone position	1.42	0.68–2.92	0.340
Patient age	1.12	1.07–1.18	<0.001
ASA	2.03	1.10–3.93	0.029
Modified MoCA	0.53	0.41–0.69	<0.001
Incision-to-suture time	1.55	1.18–2.00	0.001

ASA, American Society of Anesthesiologists; CI, confidence interval; MoCA, Montreal Cognitive Assessment.

**Table 4:** Characteristics of patients with and without postoperative delirium.

	All Patients (*n* = 760)	Patients with POD (*n* = 46)	Patients Without POD (*n* = 714)	*p*-Value
Patient age (years; median (IQR))	73.0 (67.0–80.0)	81.5 (76.2–84.8)	72.0 (67–79)	<0.001
Gender: - Male - Female	397 (52.2%) 363 (47.8%)	26 (56.5%) 20 (43.5%)	371 (52.0%) 343 (48.0%)	0.65
BMI (kg/m^2^; median (IQR))	27.0 (24.0–30.5)	26.0 (22.9–28.3)	27.0 (24.1–30.7)	0.04
ASA	I: 40 (5.3%) II: 312 (41.1%) III: 395 (52.0%) IV: 13 (1.7%)	I: 0 (0.0%) II: 8 (17.4%) III: 35 (76.1%) IV: 3 (6.5%)	I: 40 (5.6%) II: 304 (42.6%) III: 360 (50.0%) IV: 10 (1.4%)	<0.001
NYHA	I: 464 (61.1%) II: 226 (29.7%) III: 64 (8.4%) IV: 6 (0.8%)	I: 12 (26.1%) II: 23 (50.0%) III: 11 (23.9%) IV: 0 (0.0%)	I: 452 (63.3%) II: 203 (28.4%) III: 53 (7.4%) IV: 6 (0.8%)	<0.001
Modified MoCA: - 0 points - 1 points - 2 points - 3 points - 4 points - 5 points	11 (1.4%) 23 (3.0%) 53 (7.0%) 117 (15.4%) 216 (28.4%) 340 (44.7%)	3 (6.5%) 9 (19.6%) 4 (8.7%) 10 (21.7%) 12 (26.1%) 8 (17.4%)	8 (1.1%) 14 (2.0%) 49 (6.9%) 107 (15.0%) 204 (28.6%) 332 (46.5%)	<0.001
Self-care: - No problems - Few problems - Moderate problems - Many problems - Not possible	490 (64.5%) 125 (16.4%) 107 (14.1%) 35 (4.6%) 3 (0.4%)	14 (30.4%) 11 (23.9%) 16 (34.8%) 4 (8.7%) 1 (2.2%)	476 (66.7%) 114 (16.0%) 91 (12.7%) 31 (4.3%) 2 (0.3%)	<0.001
Incision-to-suture-time (minutes; median (IQR))	90.0 (60.0–133.0)	107.0 (76.0–173.0)	85.0 (60.0–130.0)	0.01

ASA, American Society of Anesthesiologists; BMI, body mass index; IQR, interquartile range; MoCA, Montreal Cognitive Assessment; NYHA, New York Heart Association; POD, postoperative delirium.

**Table 5:** Length of hospital stay and mortality rates.

	All Patients	Patients with POD	Patients Without POD	*p*-Value
LOS (days; median (IQR)): - All patients - Supine patients - Prone patients	- 7.0 (4.0–10.0) - 7.0 (4.0–10.0) - 6.0 (5.0–10.0)	14.5 (9.0–27.0) - 13.0 (9.0–29.0) - 15.5 (10.0–21.0)	7.0 (4.0–9.0) - 7.0 (4.0–10.0) - 6.0 (5.0–9.0)	<0.001 <0.001 <0.001
Mortality rate - all patients - supine patients - prone patients	- 18/760 (2.4%) - 13/549 (2.4%) - 5/211 (2.4%)	- 6/46 (13.0%) - 4/30 (13.3%) - 2/16 (12.5%)	- 12/714 (1.7%) - 9/519 (1.7%) - 3/195 (1.5%)	<0.001 <0.001 <0.001

IQR, interquartile range; LOS, length of hospital stay; POD, postoperative delirium.

## Data Availability

The original contributions presented in this study are included in the article. Further inquiries can be directed to the corresponding author.

## Abbreviations

The following abbreviations are used in this manuscript:

ASA	American Society of Anesthesiologists
BMI	Body Mass Index
CAM-ICU	Confusion Assessment Method for Intensive Care Unit
ESA	European Society of Anesthesiology
ICU	Intensive Care Unit
IQR	Interquartile Range
LOS	Length of Hospital Stay
MoCA	Montreal Cognitive Assessment
NIRS	Near-Infrared Oxygen Saturation
NYHA	New York Heart Association
POD	Postoperative Delirium
PROPDESC score	Pre-Operative Prediction of Postoperative Delirium by Appropriate Screening Score
PROPDESC-VAL-Study	Pre-Operative Prediction of Postoperative Delirium by Appropriate Screening Score
3D-CAM	3 min Diagnostic Interview for Confusion Assessment Method-Defined Delirium
